# Effects of tolvaptan discontinuation in patients with autosomal dominant polycystic kidney disease: a post hoc pooled analysis

**DOI:** 10.1186/s12882-023-03247-6

**Published:** 2023-06-22

**Authors:** Michael Lioudis, Xiaolei Zhou, Eric Davenport, Sasikiran Nunna, Holly B. Krasa, Dorothee Oberdhan, Ancilla W. Fernandes

**Affiliations:** 1 Section of Nephrology, Upstate Medical University, 343 Campus West Building (CWB), 750 East Adams Street, Syracuse, NY 13210 USA; 2 RTI Health Solutions, 3040 East Cornwallis Road, PO Box 12194, Research Triangle Park, NC 27709 USA; 3 Otsuka Pharmaceutical Development & Commercialization, Inc, 508 Carnegie Center Drive, Princeton, NJ 08540 USA; 4 Blue Persimmon Group LLC, 1701 Rhode Island Ave NW, Washington, DC 20036 USA

**Keywords:** Autosomal dominant polycystic kidney disease (ADPKD), Tolvaptan, Glomerular filtration rate, Kidney volume, Clinical trial, Treatment

## Abstract

**Background:**

Tolvaptan slows kidney function decline in patients with autosomal dominant polycystic kidney disease (ADPKD) who are at risk of rapid progression. Given that treatment requires commitment to long-term use, we evaluated the effects of tolvaptan discontinuation on the trajectory of ADPKD progression.

**Methods:**

This was a post hoc analysis of pooled data from two clinical trials of tolvaptan (TEMPO 2:4 [NCT00413777] and TEMPO 3:4 [NCT00428948]), an extension trial (TEMPO 4:4 [NCT01214421]), and an observational study (OVERTURE [NCT01430494]) that enrolled patients from the other trials. Individual subject data were linked longitudinally across trials to construct analysis cohorts of subjects with a tolvaptan treatment duration > 180 days followed by an off-treatment observation period of > 180 days. For inclusion in Cohort 1, subjects were required have ≥ 2 outcome assessments during the tolvaptan treatment period and ≥ 2 assessments during the follow-up period. For Cohort 2, subjects were required to have ≥ 1 assessment during the tolvaptan treatment period and ≥ 1 assessment during the follow-up period. Outcomes were rates of change in estimated glomerular filtration rate (eGFR) and total kidney volume (TKV). Piecewise-mixed models compared changes in eGFR or TKV in the on-treatment and post-treatment periods.

**Results:**

In the Cohort 1 eGFR population (*n* = 20), the annual rate of eGFR change (in mL/min/1.73 m^2^) was -3.18 on treatment and -4.33 post-treatment, a difference that was not significant (*P* = 0.16), whereas in Cohort 2 (*n* = 82), the difference between on treatment (-1.89) and post-treatment (-4.94) was significant (*P* < 0.001). In the Cohort 1 TKV population (*n* = 11), TKV increased annually by 5.18% on treatment and 11.69% post-treatment (*P* = 0.06). In Cohort 2 (*n* = 88), the annual TKV growth rates were 5.15% on treatment and 8.16% post-treatment (*P* = 0.001).

**Conclusions:**

Although limited by small sample sizes, these analyses showed directionally consistent acceleration in measures of ADPKD progression following the discontinuation of tolvaptan.

## Background

With the introduction of the vasopressin V2 receptor antagonist tolvaptan into clinical use in autosomal dominant polycystic kidney disease (ADPKD), a disease-specific treatment option was available for the first time for affected individuals who are at risk of rapid progression [1]. The efficacy of inhibiting vasopressin (antidiuretic hormone) signaling to slow cystic growth was demonstrated in pivotal clinical trials, in which tolvaptan-treated subjects experienced significantly slower rates of total kidney volume expansion (TEMPO 3:4 trial [NCT00428948]) and decline in kidney function (TEMPO 3:4 and REPRISE [NCT02160145] trials) compared to placebo-allocated controls [2, 3]. The efficacy, safety, and tolerability profile of tolvaptan in this patient population over the longer term have been substantiated in subsequent extension trials [4, 5].

Clinical trial data support the hypothesis that inhibition of vasopressin activity for longer periods in individuals with ADPKD exerts greater effects on the trajectory of disease progression than shorter treatment periods. The open-label, 2-year TEMPO 4:4 (NCT01214421) extension trial showed that subjects who had received tolvaptan during the preceding, 3-year, randomized TEMPO 3:4 trial continued to experience slower estimated glomerular filtration rate (eGFR) decline relative to that observed in the placebo arm of TEMPO 3:4. Consequently, the overall decline in eGFR during the 5-year period of study was smaller in participants who had received tolvaptan for 3 years in TEMPO 3:4 than those who first started treatment in TEMPO 4:4 [4].

Given the benefits of earlier treatment initiation, [4] we explored the relationship between treatment period and ADPKD outcomes from a different perspective, by assessing the effects of tolvaptan discontinuation. We performed a post hoc analysis of pooled data to evaluate the hypothesis that eGFR decline rate and total kidney volume (TKV) growth rate would worsen after discontinuation of tolvaptan.

## Methods

### Study design

**Fig. 1:**
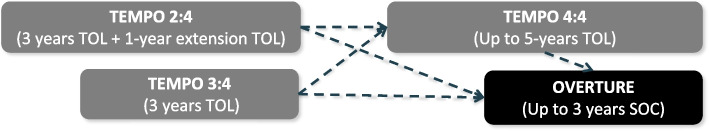
Source studies for the pooled analysis. Post-treatment follow-up and pre-treatment baseline assessments were available in TEMPO 2:4, 3:4, and 4:4 and were used as off-treatment assessments as appropriate. SOC, standard of care management not including tolvaptan treatment; TOL, tolvaptan

This study was an analysis of a pooled ADPKD database of clinical studies that was constructed as previously described [6]. For this analysis, patients were required to have ≥ 6 months of untreated follow-up after ≥ 6 months of tolvaptan therapy. As no tolvaptan clinical trials were designed for this criterion, individual subject records were linked longitudinally across tolvaptan clinical trials and an observational study to obtain subjects with the defined on-treatment and off-treatment observation periods (Fig. 1). The tolvaptan clinical trials were TEMPO 2:4 (NCT00413777), [7] TEMPO 3:4, [2] and the extension trial TEMPO 4:4 [4]. The observational study was OVERTURE (NCT01430494), [8] which enrolled tolvaptan-naïve patients as well as patients who discontinued tolvaptan clinical trials and agreed to further observation. Off-treatment assessments following clinical trial participation were available from the gap before entry into the extension trial or from the observational study. The clinical trials and observational study included in the analysis were conducted during the period 2005–2016.

For the first analysis cohort (Cohort 1), subjects were required to have a tolvaptan treatment duration > 180 days followed by an off-treatment observation period of > 180 days, and to have at least 2 outcome assessments during the tolvaptan treatment period and at least 2 assessments during the follow-up period. Because only a small number of subjects met the criteria for Cohort 1, a second analysis cohort (Cohort 2) was constructed to include additional patients with fewer assessments in the on- or off-treatment periods who would still provide at least partial data for model fitting. In Cohort 2, subjects were required to have a tolvaptan treatment duration > 180 days followed by an off-treatment observation period of > 180 days, and to have at least 1 outcome assessment during the tolvaptan treatment period and at least 1 assessment during the follow-up period. The two cohorts were created for both eGFR and for TKV. eGFR was calculated in all studies using the Chronic Kidney Disease Epidemiology Collaboration (CKD-EPI) equation [9]. TKV was measured by magnetic resonance imaging.

### Statistical analyses

For the outcome analyses, eGFR assessments collected during the first week of treatment and during the first week after treatment were excluded due to the acute and reversible hemodynamic effect of tolvaptan [10, 11]. TKV assessments that occurred < 7 days prior to the first dose date and < 7 days after the last dose date were considered as on-treatment because of infrequent TKV assessments.

Patient demographics, baseline disease characteristics, tolvaptan use prior to study start, on-treatment duration (in years), tolvaptan dose, and off-tolvaptan follow-up (in years) were summarized descriptively for each analysis cohort. Postbaseline eGFR and TKV assessments of Cohort 1 were plotted over time for individual patient profiles. Assessments were aligned based on the last dose date of tolvaptan, which allowed for easy visual comparison between the on- and off-tolvaptan periods.

Piecewise-mixed models, as described previously, [6] were used to compare changes in eGFR or TKV in the on-treatment and off-treatment periods. For eGFR, the piecewise-mixed model included a random intercept to account for the within-subject correlation and fixed effects for years on treatment, years off treatment, baseline eGFR, and the reversal of the hemodynamic effect following tolvaptan discontinuation (indicator variable: 1 = assessment occurred off-tolvaptan treatment; 0 = otherwise). For TKV, the log transformation was applied to assessment values due to their exponential increase over time. The piecewise-mixed model included a random intercept to account for the within-subject correlation and fixed effects for years on treatment, years off treatment, and baseline TKV (in logarithm).

## Results

### Baseline characteristics, tolvaptan exposure, and follow-up

Cohort 1 for eGFR analysis comprised 20 patients, of whom 70% were female and 95% were White (Table 1). The mean (standard deviation [SD]) baseline eGFR was 84 (26) mL/min/1.73 m^2^. Of the 20 patients, 50% reported having stage G1 chronic kidney disease; another 25% reported having stage G2 disease and 25% stage G3; 40% were in ADPKD risk classification (i.e., Mayo class) 1D, 25% in 1C, and 25% in 1E. None had prior exposure to tolvaptan (Table 2). Over the study analysis period, the patients averaged 2.2 years on treatment and 3.6 years off treatment, with a mean (SD) tolvaptan dose of 81.6 (28.2) mg/day (range: 42.1 to 119.3 mg/day). The mean (SD) number of eGFR assessments per subject was 8 (3.5) while on treatment and 6 (2.2) post-treatment.

**Table 1:** Patient baseline characteristics

		**Cohort 1** ** ≥ 2 on treatment, ≥ 2 post-treatment assessments**		**Cohort 2** ** ≥ 1 on treatment, ≥ 1 post-treatment assessments**	
**Characteristic**	**Statistic or Category**	**eGFR Analysis** **(*n* = 20)**	**TKV Analysis** **(*n* = 11)**	**eGFR Analysis** **(*n* = 82)**	**TKV Analysis** **(*n* = 88)**
Age, years	Mean (SD)	39.9 (8.5)	40.9 (8.1)	40.3 (7.4)	40.7 (7.2)
	Median	41.2	41.3	41.1	41.5
	Min, Max	23.6, 50.2	23.6, 50.2	23.6, 52.1	23.6, 51.9
Sex	Male	6 (30%)	3 (27%)	40 (49%)	43 (49%)
	Female	14 (70%)	8 (73%)	42 (51%)	45 (51%)
Race	White	19 (95%)	11 (100%)	76 (93%)	80 (91%)
	Black	1 (5%)	0	3 (4%)	4 (5%)
	Hispanic	0	0	3 (4%)	4 (5%)
	Other	0	0	0	0
Height, m	Mean (SD)	1.72 (0.11)	1.72 (0.11)	1.73 (0.11)	1.74 (0.11)
Weight, kg	Mean (SD)	77.1 (16.6)	77.7 (18.3)	78.3 (16.9)	79.5 (17.3)
Body mass index, kg/m^2^	Mean (SD)	25.8 (3.5)	25.9 (3.3)	26.0 (4.3)	26.2 (4.6)
Age at ADPKD diagnosis, years	Mean (SD)	27.7 (9.6)	25.5 (8.2)	27.3 (9.3)	27.6 (9.1)
Chronic kidney disease stage, mL/min/1.73 m^2^	≥ 90 (stage G1)	10 (50%)	5 (50%)	29 (35%)	22 (25%)
	60 to < 90 (stage G2)	5 (25%)	1 (10%)	32 (39%)	37 (43%)
	30 to < 60 (stage G3)	5 (25%)	4 (40%)	21 (26%)	28 (32%)
	< 30 (stage G4)	0	0	0	0
Baseline eGFR, mL/min/1.73 m^2^	Mean (SD)	84 (26)	82 (27)	80 (22)	75 (22)
	Min, Max	44, 122	45, 116	36, 126	34, 126
Systolic blood pressure, mmHg	Mean (SD)	127.0 (13.1)	125.0 (11.6)	125.4 (11.6)	124.7 (11.7)
Diastolic blood pressure, mmHg	Mean (SD)	80.3 (9.6)	81.2 (11.2)	79.6 (7.9)	79.8 (8.7)
TKV, mL	Mean (SD)	2109 (1467)	1828 (792)	1876 (1120)	1897 (1074)
	Min, Max	836, 6958	873, 3436	572, 6958	416, 6958
ADPKD risk classification	Class 1A	0	0	0	1 (1%)
	Class 1B	2 (10%)	1 (9%)	10 (12%)	8 (9%)
	Class 1C	5 (25%)	3 (27%)	25 (31%)	32 (36%)
	Class 1D	8 (40%)	4 (36%)	31 (38%)	31 (35%)
	Class 1E	5 (25%)	3 (27%)	16 (20%)	16 (18%)

*ADPKD* autosomal dominant polycystic kidney disease, *eGFR* estimated glomerular filtration rate, *Max* maximum, *Min* minimum, *SD* standard deviation, *TKV* total kidney volume

**Table 2:** Tolvaptan exposure and follow-up

		**Cohort 1** ** ≥ 2 on treatment, ≥ 2 post-treatment assessments**		**Cohort 2** ** ≥ 1 on treatment, ≥ 1 post-treatment assessments**	
**Characteristic**	**Statistic or Category**	**eGFR Analysis** **(*n* = 20)**	**TKV Analysis** **(*n* = 11)**	**eGFR Analysis** **(*n* = 82)**	**TKV Analysis** **(*n* = 88)**
Prior exposure to tolvaptan	Yes	0	0	4 (5%)	16 (18%)
	No	20 (100%)	11 (100%)	78 (95%)	72 (82%)
Treatment duration, years	Mean (SD)	2.2 (1.1)	3.1 (0.3)	2.8 (0.7)	3.1 (0.7)
	Min, Max	0.6, 3.9	2.8, 3.9	0.6, 4.4	1.0, 4.5
Duration of post-tolvaptan follow-up, years	Mean (SD)	3.6 (1.1)	2.1 (0.5)	1.5 (1.3)	1.2 (0.8)
	Min, Max	1.9, 5.1	1.3, 2.9	0.5, 5.1	0.5, 4.6
Average daily tolvaptan dose, mg/day^a^	Mean (SD)	81.6 (28.2)	83.7 (25.6)	97.4 (25.1)	93.0 (26.1)
	Median	73.7	80.6	112.6	101.1
	Q1, Q3	55.9, 118.4	60.3, 119.1	80.6, 119.1	62.2, 118.0
	Min, Max	42.1, 119.3	54.7, 119.3	42.1, 119.6	42.1, 119.6
Completion/termination status	Completed	7 (35%)	8 (73%)	69 (84%)	78 (89%)
	Discontinued treatment early	13 (65%)	3 (27%)	13 (16%)	10 (11%)
Reason for discontinuation^b^	Adverse events	8 (62%)	1 (33%)	8 (62%)	6 (60%)
	Subject met withdrawal criteria	1 (8%)	0	1 (8%)	0
	Subject withdrew consent	4 (31%)	2 (67%)	4 (31%)	4 (40%)
Number of on-treatment eGFR assessments	Mean (SD)	8 (3.5)		10 (2.6)	
	Median	9		10	
	Q1, Q3	5, 10		9, 10	
	Min, Max	2, 15		2, 16	
Number of post-treatment eGFR assessments	Mean (SD)	6 (2.2)		4 (1.6)	
	Median	6		4	
	Q1, Q3	5, 7		4, 4	
	Min, Max	2, 13		1, 13	
Number of on-treatment TKV assessments	Mean (SD)		3 (0.3)		3 (1.6)
	Median		3		3
	Q1, Q3		3, 3		3, 3
	Min, Max		3, 4		1, 7
Number of post-treatment TKV assessments	Mean (SD)		2 (0.4)		1 (0.6)
	Median		2		1
	Q1, Q3		2, 2		1, 2
	Min, Max		2, 3		1, 3

*eGFR *estimated glomerular filtration rate, *Max *maximum, *Min *minimum, *Q1 *first quartile, *Q3 *third quartile, *SD* standard deviation, *TKV *total kidney volume

^a^Average daily dose was calculated as the total actual dose divided by the duration of treatment

^b^Percentages were calculated using the number of subjects who discontinued treatment early as the denominator

Cohort 1 for TKV analysis comprised 11 patients with mean (SD) baseline TKV of 1828 (792) mL (Table 1). The mean (SD) number of TKV assessments per subject was 3 (0.3) while on treatment and 2 (0.4) post-treatment.

Cohort 2 for eGFR consisted of 82 patients, of whom 51% were female and 93% were White (Table 1). The mean (SD) baseline eGFR was 80 (22) mL/min/1.73 m^2^. Most patients had stage G1 or G2 chronic kidney disease (74%), and 26% had stage G3; 38% were in ADPKD risk classification 1D, 31% were in 1C, and 20% were in 1E. Only 4 of the 82 patients (5%) had prior exposure to tolvaptan (Table 2). Over the study analysis period, this group averaged 2.8 years on treatment and 1.5 years off treatment, at a mean (SD) tolvaptan dose of 97.4 (25.1) mg/day (range: 42.1 to 119.6 mg/day). The mean (SD) number of eGFR assessments per subject was 10 (2.6) while on treatment and 4 (1.6) post-treatment.

Cohort 2 for TKV comprised 88 patients with mean (SD) baseline TKV of 1897 (1074) mL (Table 1). The mean (SD) number of TKV assessments per subject was 3 (1.6) while on treatment and 1 (0.6) post-treatment.

### Patient profile plots

**Fig. 2:**
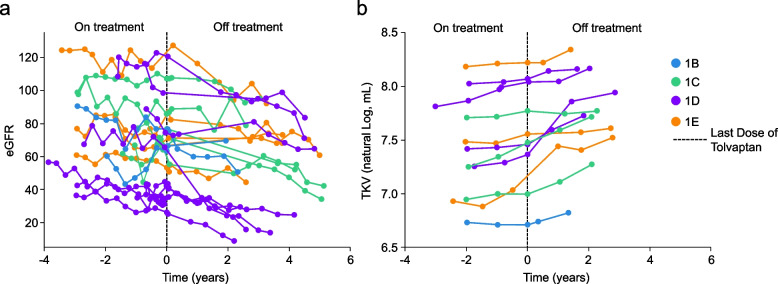
Plots of **a**) eGFR and **b**) TKV over time for individual patients in Cohort 1. Patients are designated by color as Mayo risk class 1B–1E. **a** eGFR collected during the first week of treatment and first week posttreatment was removed. **b** Data points shown in the figure prior to time 0 (last dose of tolvaptan) were on-treatment assessments. Baseline assessments that were prior to the first dose of tolvaptan are not shown in the figure. eGFR, estimated glomerular filtration rate; TKV, total kidney volume

For eGFR in Cohort 1, patient profile plots showed a slight worsening of kidney function overall while on treatment. Although a few patients experienced increases in eGFR (i.e., improvement in kidney function) at some timepoints during the off-tolvaptan period, kidney function for patients overall worsened when compared with the on-tolvaptan period (Fig. 2a). For TKV in Cohort 1, patient profile plots for TKV showed a pattern of stabilization or slight increase in logarithm values of TKV during the on-tolvaptan period. During the off-tolvaptan period, most kidney volumes increased, although a few patients maintained their kidney volume levels (Fig. 2b).

### Comparison of on-treatment and off-treatment changes in eGFR and TKV

**Fig. 3:**
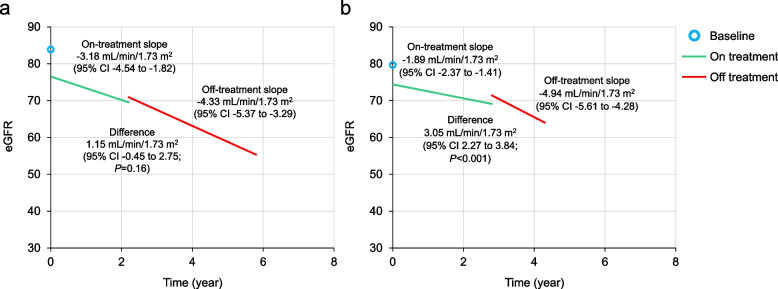
Decline in eGFR during the on-treatment and off-treatment periods. **a** Cohort 1; **b** Cohort 2. The estimations were calculated from the piecewise-mixed model using the mean baseline eGFR (shown as a blue circle at time 0) in the analysis set. The estimations during the on-treatment period (the green line segment) started at the first postbaseline assessment and ended at the mean duration of the tolvaptan treatment. For the off-treatment period (the red line segment), the estimations were calculated for the mean follow-up (off-treatment) time. The gap between the green and red line segments reflected the estimated reverse of hemodynamic effect following tolvaptan discontinuation. CI, confidence interval; eGFR, estimated glomerular filtration rate

In Cohort 1, the estimated annual rate of change (the slope) of eGFR in the piecewise mixed model was -3.18 mL/min/1.73 m^2^ (95% confidence interval [CI] -4.54 to -1.82) while on treatment and was -4.33 mL/min/1.73 m^2^ (95% CI -5.37 to -3.29) while off treatment (Fig. 3a). The difference in slopes between the on-tolvaptan and the off-tolvaptan periods was 1.15 mL/min/1.73 m^2^ (95% CI: -0.45 to 2.75), which was not statistically significant (*P* = 0.16). In Cohort 2, estimated annual change rate in eGFR was -1.89 mL/min/1.73 m^2^ (95% CI -2.37 to -1.41) during the on-tolvaptan period and -4.94 mL/min/1.73 m^2^ (95% CI -5.61 to -4.28) during the off-tolvaptan period (Fig. 3b). The difference in slopes of 3.05 mL/min/1.73 m^2^ (95% CI 2.27–3.84) was statistically significant (*P* < 0.001). Subgroups within Cohort 2 defined by greater or lesser risk class or sex consistently showed worsening of eGFR decline after treatment discontinuation (Table 3).

**Table 3:** Annual rates of change in eGFR (mL/min/1.73 m^2^, 95% CI) for subgroups of Cohort 2

	**Number of subjects**	**On treatment**	**Post-treatment**	**Difference**
Risk class 1B or 1C	1B: 10; 1C: 25	-1.02 (-1.77, -0.26)	-5.05 (-6.22, -3.88)	4.03 (2.70, 5.36), *P* < 0.001
Risk class 1D or 1E	1D: 31; 1E: 16	-2.66 (-3.29, -2.04)	-4.84 (-5.63, -4.05)	2.17 (1.21, 3.13), *P* < 0.001
Female	42	-1.59 (-2.32, -0.87)	-5.47 (-6.36, -4.58)	3.88 (2.80, 4.96), *P* < 0.001
Male	40	-2.22 (-2.85, -1.59)	-3.63 (-4.72, -2.53)	1.41 (0.19, 2.62), *P* = 0.02

*CI* confidence interval, *eGFR* estimated glomerular filtration rate

**Fig. 4:**
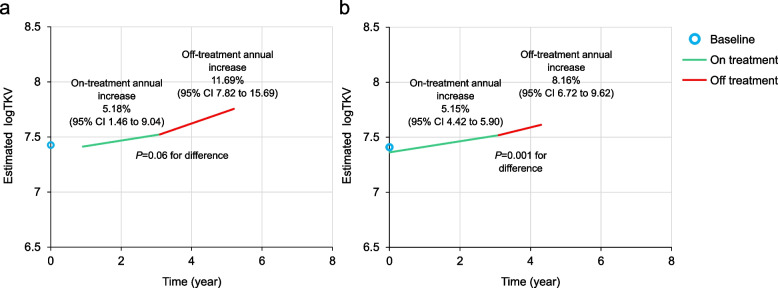
Estimated logarithm of TKV during the on-treatment and off-treatment periods. **a** Cohort 1; **b** Cohort 2. The estimations were calculated from the piecewise-mixed model using the mean baseline log TKV (shown as a blue circle at time 0) in the analysis set. The estimations during the on-treatment period (the green line segment) started at the first postbaseline assessment and ended at the mean duration of the tolvaptan treatment. For the off-treatment period (the red line segment), the estimations were calculated for the mean follow-up (off-treatment) time. TKV, total kidney volume

In Cohort 1, the estimated annual percentage increase in TKV for the on-tolvaptan period was 5.18% (95% CI 1.46 to 9.04) and for the off-treatment period was 11.69% (95% CI 7.82 to 15.69; Fig. 4a). The difference approached statistical significance (*P* = 0.06). In Cohort 2, the estimated annual percentage increase in TKV during the on-tolvaptan period was 5.15% (95% CI 4.42 to 5.90) and during the off-tolvaptan period was 8.16% (95% CI 6.72 to 9.62) (Fig. 4b). The difference was statistically significant (*P* = 0.001).

## Discussion

This pooled database analysis assessed trajectories of TKV growth and eGFR decline over long-term follow-up during tolvaptan treatment and after discontinuation. Subjects in the two analysis cohorts had a mean duration of tolvaptan treatment ranging from 2.2 to 3.1 years and off-treatment follow-up ranging from 1.2 to 3.6 years. The analysis population was drawn from clinical trials and reflected enrollment populations at increased risk of rapid progression.

Results of the piecewise mixed model suggested worsening of the eGFR decline rate after treatment discontinuation, with directional consistency between both analysis cohorts. Although the difference between on treatment and post-treatment periods in Cohort 1 was not significant, it was significant in Cohort 2, a larger analysis set (including additional subjects with fewer on/off-treatment assessments) and with higher mean tolvaptan dose. The decline in eGFR also accelerated post-treatment across Cohort 2 subgroups defined by ADPKD risk class and sex. The post-treatment rates of decline were not faster in the subgroup with worse risk class (1D or 1E) than in that with lesser risk class (1B or 1C), as would be expected, again possibly due to the small sizes of the subgroups.

Similar results were obtained for the TKV analyses, which showed worsening in TKV growth after treatment discontinuation, with directional consistency between Cohort 1 and Cohort 2 and a significant difference between on-treatment and off-treatment rates of change in the larger cohort. Results of modeling were corroborated by visual plotting of changes in eGFR and TKV.

Although conclusions about the differences between on treatment and post-treatment rates of change are limited by sample sizes, it is notable that the on treatment annual rate of change in eGFR in the cohort with the most eGFR assessment data (Cohort 1; -3.18 mL/min/1.73 m^2^) is similar in magnitude to the annual rates in tolvaptan-treated subjects in TEMPO 3:4 (-2.72 mL/min/1.73 m^2^) and TEMPO 4:4 (-3.26 mL/min/1.73 m^2^ for those who started tolvaptan treatment in TEMPO 3:4 and -3.14 mL/min/1.73 m^2^ for those who started treatment in TEMPO 4:4) [2, 4]. The slope off treatment in Cohort 1 (-4.33 mL/min/1.73 m^2^) was also similar to that in placebo-allocated subjects in TEMPO 3:4 (-3.70 mL/min/1.73 m^2^) [2].

The results reported here are consistent with data from a tolvaptan-treated cohort with 3 years of follow-up, in which treatment discontinuation was associated with attenuated eGFR benefit relative to continuation for the full duration of follow-up [7]. These findings are supported by the short-term effects of discontinuing tolvaptan, which is taken in a daily split-dose regimen to provide 24-h suppression of vasopressin activity. Following discontinuation, the aquaretic effects of treatment rapidly disappear, suggesting cessation of effects on vasopressin signaling [11]. Accordingly, vasopressin-driven pathomechanisms would be expected to resume, with a return to untreated rates of ADPKD progression.

Given the long-term use of tolvaptan following treatment initiation in ADPKD, situations may arise in which patients may need or want to interrupt therapy, for example, before an anticipated pregnancy, in situations in which maintenance of adequate hydration is impossible (e.g., before surgery), or for situations in which polyuria would be disruptive [1, 12]. The results of this analysis, which indicate that ADPKD returns to more rapid rates of progression after tolvaptan discontinuation, provide information to help healthcare providers and patients weigh the risks and benefits of treatment interruptions.

Limitations of the analysis include its retrospective design and the small sample sizes. Additionally, most of the sample was in CKD stages G1-G2, with none in G4 or greater or aged > 55 years. The findings are thus less likely to be applicable to patients in later-stage ADPKD. Patients in Cohort 2 were required to have only 1 post-treatment eGFR assessment, which must be considered a limitation given that renal function estimation is a dynamic parameter subject to fluctuations [13]. Although most patients in Cohort 2 had multiple post-treatment eGFR assessments (median = 4, first quartile = 4), some did not (range of 1 to 13 assessments).

## Conclusions

Results of this post hoc analysis indicate that following treatment discontinuation in patients with ADPKD, suppression of eGFR decline and TKV growth by tolvaptan ceases and disease progression accelerates. Continuation of therapeutic benefit is dependent on maintenance of inhibition of the vasopressin-driven pathway of cystic expansion. Individualized treatment decision-making based on anticipated risks and benefits will enable patients to make well-informed decisions regarding treatment interruption in collaboration with their healthcare providers.

## Data Availability

To submit inquiries related to Otsuka clinical research, or to request access to individual participant data (IPD) associated with any Otsuka clinical trial, please visit https://clinical-trials.otsuka.com/. For all approved IPD access requests, Otsuka will share anonymized IPD on a remotely accessible data sharing platform.

## Abbreviations

ADPKD: Autosomal dominant polycystic kidney disease
CI: Confidence interval
CKD-EPI: Chronic Kidney Disease Epidemiology Collaboration
eGFR: Estimated glomerular filtration rate
SD: Standard deviation
TKV: Total kidney volume
